# Effect of Inhaled Carbon Ultrafine Particles on Reactive Hyperemia in Healthy Human Subjects

**DOI:** 10.1289/ehp.10323

**Published:** 2007-12-17

**Authors:** Alpa P. Shah, Anthony P. Pietropaoli, Lauren M. Frasier, Donna M. Speers, David C. Chalupa, Joseph M. Delehanty, Li-Shan Huang, Mark J. Utell, Mark W. Frampton

**Affiliations:** 1 Department of Medicine; 2 Department of Biostatistics and; 3 Department of Environmental Medicine, University of Rochester Medical Center, Rochester, New York, USA

**Keywords:** air pollution, nitric oxide, particulate matter, reactive hyperemia, vascular

## Abstract

**Background:**

Ultrafine particles (UFP) may contribute to the cardiovascular effects of exposure to particulate air pollution, partly because of their relatively efficient alveolar deposition and potential to enter the pulmonary vascular space.

**Objectives:**

This study tested the hypothesis that inhalation of elemental carbon UFP alters systemic vascular function.

**Methods:**

Sixteen healthy subjects (mean age, 26.9 ± 6.5 years) inhaled air or 50 μg/m^3^ elemental carbon UFP by mouthpiece for 2 hr, while exercising intermittently. Measurements at preexposure baseline, 0 hr (immediately after exposure), 3.5 hr, 21 hr, and 45 hr included vital signs, venous occlusion plethysmography and reactive hyperemia of the forearm, and venous plasma nitrate and nitrite levels.

**Results:**

Peak forearm blood flow after ischemia increased 3.5 hr after exposure to air but not UFP (change from preexposure baseline, air: 9.31 ± 3.41; UFP: 1.09 ± 2.55 mL/min/100 mL; *t*-test, *p* = 0.03). Blood pressure did not change, so minimal resistance after ischemia (mean blood pressure divided by forearm blood flow) decreased with air, but not UFP [change from preexposure baseline, air: −0.48 ± 0.21; UFP: 0.07 ± 0.19 mmHg/mL/min; analysis of variance (ANOVA), *p* = 0.024]. There was no UFP effect on pre-ischemia forearm blood flow or resistance, or on total forearm blood flow after ischemia. Venous nitrate levels were significantly lower after exposure to carbon UFP compared with air (ANOVA, *p* = 0.038). There were no differences in venous nitrite levels.

**Conclusions:**

Inhalation of 50 μg/m^3^ carbon UFP during intermittent exercise impairs peak forearm blood flow during reactive hyperemia in healthy human subjects.

Cardiovascular morbidity and mortality are associated with exposure to air pollution particles (Bremner et al. 1999; Burnett et al. 1999; Peters et al. 2000, 2001; Pope et al. 2004). Data analyzed from 1.2 million adults in the United States from 1982–1998 linked 10-μg/m^3^ elevations in particulate matter (PM) < 10 μm in diameter (PM_10_) with a 6% increase in deaths from cardiopulmonary disease (Pope et al. 2002). Among > 65,000 women followed in the Women’s Health Initiative study (Miller et al. 2007), a 10-μg/m3 increase in PM < 2.5 μm (PM=_2.5_) was associated with a 24% increased risk for a cardiovascular event, and a 76% increase in the risk of death from cardiovascular disease. Exposure to PM has been associated with increased risk for myocardial infarction (Peters et al. 2001, 2004), cardiac arrhythmias (Peters et al. 2000), increases in blood pressure (Ibald-Mulli et al. 2001), reductions in heart rate variability (Gold et al. 2000; Pope et al. 1999), increased blood markers of inflammation and coagulation (Rückerl et al. 2006), and more rapid progression of atherosclerosis (Künzli et al. 2005). Despite convincing epidemiologic evidence linking particle exposure and cardiovascular disease, the relevant particle characteristics and mechanisms remain poorly defined.

Ultrafine particles (UFP) (diameter < 100 nm) have been hypothesized as contributors to cardiovascular effects of PM. High concentrations of UFP are found on and near major highways (Kittelson et al. 2004), where they may contribute to the increased risk of myocardial infarction associated with exposure to traffic (Peters et al. 2004). Their characteristics suggest that UFP may contribute to the cardiovascular effects of PM. Compared with fine particles at similar mass concentrations, UFP have a higher number concentration and surface area (Oberdörster et al. 1995), enhanced oxidant capacity (Brown et al. 2001; Li et al. 2003), greater inflammatory potential (Oberdörster et al. 1995), and higher pulmonary deposition efficiency (Chalupa et al. 2004; Daigle et al. 2003). They are also more likely to penetrate epithelium and enter the pulmonary interstitium and vascular space (Stearns et al. 1994).

We have previously reported that exposure to 50 μg/m^3^ UFP caused a significant decrease in the pulmonary diffusion capacity for carbon monoxide 21 hr after exposure (Pietropaoli et al. 2004b), as well as changes in blood leukocyte expression of surface adhesion molecules (Frampton et al. 2006). These findings together suggest that inhalation of carbon UFP caused transient reductions in the pulmonary capillary blood volume, providing indirect evidence for effects of UFP on pulmonary endothelial function.

In this study, we describe the effects of inhaled UFP on a measure of systemic vascular function that is associated with coronary artery disease, forearm venous plethysmography, and reactive hyperemia (RH). We hypothesized that inhalation of elemental carbon UFP impairs RH in healthy nonsmokers. Plasma concentrations of nitrite and nitrate, products of nitric oxide metabolism, were measured to determine whether impairment in vasorelaxation was accompanied by alterations in NO metabolism. Some data from this study have been presented previously in abstract form (Pietropaoli et al. 2004a).

## Materials and Methods

### Subjects

The study was approved by the Research Subjects Review Board at the University of Rochester, and subjects provided written informed consent. We studied 16 healthy never-smokers (eight male and eight female) 18–40 years of age. Subjects were required to have no history of pulmonary or cardiovascular disease, normal spirometry, a normal electrocardiogram, and a negative urine pregnancy test (females). Subjects were instructed to exclude anti-inflammatory drugs for the duration of the study and to exclude caffeine, large fatty meals, and vigorous exercise during and 24 hr before study days. Subjects were not studied within 6 weeks of a respiratory infection.

### Study design

The study used a double-blinded, randomized (blocked by order of presentation and sex), crossover design.

Subjects underwent a 2-hr mouthpiece exposure to either filtered air or carbon UFP, with four 15-min exercise periods on a bicycle ergometer (target minute ventilation 20 L/min/m^2^ body surface area). A 10-min break off the mouthpiece was taken after 1 hr of exposure. Measurements (vital signs, RH, and phlebotomy) were repeated immediately (0 hr), 3.5 hr, 21 hr, and 45 hr after exposure. Exposures were separated by at least 3 weeks.

### Exposure system

Exposures were undertaken within an environmental chamber, using a mouthpiece exposure system. Briefly, the design was a one-pass, dynamic-flow exposure system. The particles [count median diameter ∼25 nm; geometric standard deviation (GSD) ∼1.6] were generated in an argon atmosphere using an electric spark discharge between two graphite electrodes, in a commercial generator (Palas Co., Karlsruhe, Germany) that had been modified to prevent any off-gassing of organic materials from within the generator (McDonald et al. 2001). This produced particles consisting of > 95% elemental carbon, without metals. The particles were then deionized and diluted with filtered air to the desired concentration. Particles were continuously generated, and the exposure concentration was monitored and regulated during the exposure. Particle number, mass, and size distributions were monitored on both the inspiratory and expiratory sides of the subject. The subject inhaled from a mouthpiece and wore a nose clip. One-way valves (Hans-Rudolph, Inc., Kansas City, MO) prevented rebreathing of UFP. Air for the control exposures and for dilution of the particles was passed through charcoal and high-efficiency particle filters and was essentially free of particles (0–10 particles/cm^3^). The rationale and design of the exposure facility have been described in detail elsewhere (Chalupa et al. 2002).

### Reactive hyperemia and nitric oxide metabolites

We measured RH of the forearm by venous occlusion plethysmography using the methodology reported by Delehanty et al. (1999). After subjects had a light breakfast, RH was measured supine in a quiet room, at the same time of day for each exposure, and before any other measurements such as phlebotomy and spirometry, because these studies may influence the measurements. To minimize dietary influence on plasma nitrate levels, subjects were placed on a low-nitrate diet on study days. A venous occlusion cuff was placed around the arm just above the elbow with the subject in the supine position, with the forearm at or above the level of the right atrium. A 1-min ischemic occlusion was performed for preconditioning. Three minutes later, a pediatric blood pressure cuff was positioned around the wrist to exclude wrist flow artifact, and a silicone strain gauge (Hokanson EC 5R; D.E. Hokanson, Inc., Bellevue, WA) was positioned so that it encircled the forearm at its widest diameter. With the wrist cuff inflated, the upper arm cuff was inflated to 40 mmHg and recordings of impedance were made for 5 sec. Five cycles of venous flow occlusion and 10 sec of cuff deflation were performed, and the results were averaged. Transient arterial occlusion was produced by inflating an upper arm cuff to 50 mmHg above systolic pressure for 5 min. Immediately after release of the occluding cuff, measurements of flow were obtained at 15-sec intervals for 3 min. Peak flow was defined as the maximum flow rate after release of the cuff. Minimal resistance was the mean arterial blood pressure divided by the peak flow. Total flow was the area under the 3-min flow curve.

We measured NO metabolites using the Sievers 270B NO analyzer (GE Analytical Instruments, Boulder, CO) equipped with a purge vessel. We measured the sum of plasma nitrite and nitrate by reducing nitrate to nitrite via nitrate reductase (Roche Diagnostics Corporation, Indianapolis, IN). The resulting nitrite was then further reduced to NO with potassium iodide in the purge vessel, and quantified by the NO analyzer. Injection of untreated plasma into the potassium iodide in the purge vessel measured plasma nitrite alone. We then calculated plasma nitrate concentrations by subtracting plasma nitrite from the sum of nitrite and nitrate to provide nitrate concentrations. Standard curves were generated using known concentrations of sodium nitrate and sodium nitrite (1.56–100 uM), and were used to verify the efficacy of the nitrate reductase. Data were acquired and analyzed using a Macintosh G4 computer with MacLab software (ADInstruments, Cupertino, CA). The lower limit of nitrate detection was 1.56 μM.

### Data handling and statistical methods

We used a standard, two-period crossover design in which each subject received both particles and air (Pietropaoli et al. 2004b). The order of presentation was randomized separately for each sex, with half of the subjects receiving each of the two possible orders. There was a washout period of 3 weeks between the two exposures to avoid the possibility of carryover effects. Our approach is based on using mixed models for the analysis of crossover trials with repeated measurements within treatment (exposure) periods (Jones and Kenward 2003). Order of presentation and sex were treated as between-subject factors, and treatment, period, and time (when repeated measurements were made after each exposure) were considered within-subject factors. We performed the approximate *t*-tests and *F*-tests (Verbeke and Molenberghs 2000) comparing UFP versus filtered air exposure and evaluating significance of other factors (e.g., time). A *p* < 0.05 was considered statistically significant.

## Results

### Subjects and UFP characteristics

We studied 16 subjects, 8 male and 8 female, with mean (±SD) age 26.9 ± 6.5 years. The mean (± SD) UFP exposure parameters were as follows: target mass concentration of 50 μg/m^3^, with a measured mass concentration of 50.0 ± 3.9 μg/m^3^, particle number concentration 10.8 ± 1.7 ×10^6^ particles/cm^3^, and particle count median diameter 27.9 ± 2.2 nm, with a GSD of 1.65 ± 0.02.

### Heart rate, blood pressure, and oxygen saturation

**Figure 1:**
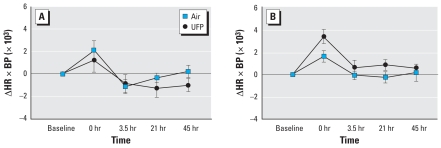
Change in the product of HR ×BP (mmHg) in females (*A*) and males (*B*). Data are mean ± SE. UFP × sex, *p* = 0.029.

There were no effects of UFP exposure on oxygen saturation, heart rate, or blood pressure (Table 1). The analysis of variance (ANOVA) revealed a significant interaction between sex and exposure for the heart-rate–systolic-blood-pressure product (HR ×BP), an indicator of cardiac work (*p* = 0.029). Males but not females showed a greater increase in HR ×BP immediately after exposure to UFP than to air (Figure 1). There were no other convincing sex-specific responses.

### Forearm blood flow and reactive hyperemia

**Figure 2:**
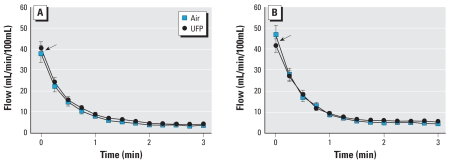
Forearm blood flow after ischemia, 0 hr (*A*) and 3.5 hr (*B*) after exposure. Data are mean ± SE. Arrows indicate peak flow, which increased 3.5 hr after air but not UFP exposure. There were no significant differences between air and UFP exposure for total flow (area under the curve).

**Figure 3:**
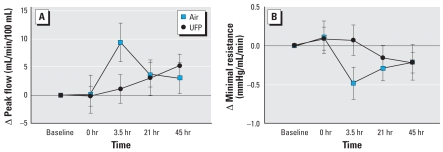
Changes from preexposure baseline in peak flow (*A*) and minimal resistance (*B*) at all time points. Data are mean ± SE. Peak flow increased, and minimal resistance decreased, 3.5 hr after air but not UFP exposure. The difference in peak flow 3.5 hr after exposure was significant by paired *t*-test (*p* = 0.034) but not by ANOVA (*p* = 0.143). The difference in minimal resistance was significant by ANOVA (UFP × time, *p* = 0.024).

Figure 2 shows the forearm blood flow after ischemia at 0 and 3.5 hr after exposure to air and UFP. Time 0 represents the peak flow measured on release of the occluding cuff. At 3.5 hr after exposure (Figure 2B), a small reduction in peak flow was seen with UFP relative to air exposure, with no differences for the subsequent measurements. Figure 3 shows the peak flow and minimal resistance as changes from preexposure baseline at all time points. Peak flow increased, and minimal resistance decreased, 3.5 hr after exposure to air. This increase in peak flow was not seen with UFP exposure. The difference in peak flow at this time point was significant by crossover paired *t*-test [change from preexposure baseline, air: 9.31 ± 3.41; UFP: 1.09 ± 2.55 mL/min/100 mL; *p* = 0.034 (Figure 3A)] but there was no difference across the entire 48-hr measurement period by ANOVA (exposure–time interaction, *p* = 0.143). The difference in minimal resistance was significant by ANOVA [change from preexposure baseline, air: −0.48 ± 0.21; UFP: 0.07 ± 0.19 mmHg/mL/min; exposure–time interaction, *p* = 0.024 (Figure 3B)]. There was no UFP effect on baseline flow, baseline resistance, or total flow.

### Nitrite and nitrate

**Figure 4:**
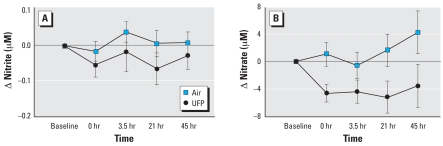
Venous plasma concentrations of nitrite (*A*) and nitrate (*B*). Data are mean ± SE. Both nitrite and nitrate levels decreased after UFP exposure; the changes in nitrate were significant by ANOVA (*p* = 0.038).

**Figure 5:**
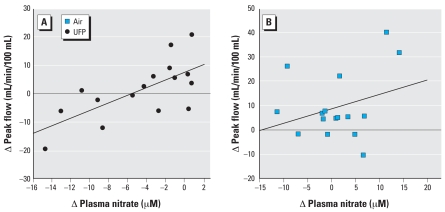
Relationship between peak forearm blood flow (change from pre-UFP exposure to 3.5 hr after exposure) and plasma nitrate concentrations (change from pre-UFP exposure to 0 hr after exposure). (*A*) UFP (*r* = 0.69, *p* = 0.003). (*B*) Air (*r* = 0.30, *p* = 0.26). There was no significant relationship between forearm blood flow and nitrate levels after air exposure.

Venous nitrate levels decreased after UFP exposure, and were significantly lower than after air exposure [UFP: 29.61 ± 3.41 μM; air: 32.52 ± 2.79 μM 21 hr after exposure, *p* = 0.038 by ANOVA (Figure 4B)]. Venous nitrite levels also decreased, but the changes were not statistically significant (*p* = 0.286). The changes in peak RH 3.5 hr after UFP but not air exposure were significantly correlated with the changes in plasma nitrate levels immediately after exposure (Figure 5), but not with changes in nitrate levels at subsequent measurements. There were no significant relationships between changes in RH and BP ×HR.

## Discussion

These are the first human experimental studies examining the systemic vascular effects of breathing an aerosol of pure UFP. The particles consisted of elemental carbon, without organic compounds or metals that have been implicated in PM effects. We tested the hypothesis that inhalation of carbon UFP impairs forearm RH in healthy nonsmokers. Peak hyperemic flow during RH increased, and minimal vascular resistance decreased, 3.5 hr after air exposure with intermittent exercise. Breathing UFP blunted these changes (Figure 2). There was no significant effect of UFP on total forearm flow during RH. UFP exposures significantly reduced venous nitrate concentrations.

We also found an increase in HR ×BP in males after UFP exposure [exposure–sex interaction, *p* = 0.03 (Figure 1)]. Hormonal influences may have protected the females from this effect. HR ×BP, or the rate–pressure product, is a well-recognized noninvasive estimate of myocardial work, although the cardiac pressure–volume loop is the gold standard for assessing contractility. Changes in HR ×BP predict myocardial oxygen consumption during exercise (Gobel et al. 1978), and may reflect changes in cardiac function or peripheral vascular tone. Not all changes in cardiac oxygen consumption are reflected by HR ×BP. However, increases in HR ×BP would be expected to worsen myocardial ischemia in patients with critical coronary artery disease. Some studies have observed increases in blood pressure associated with exposure to ambient PM (Ibald-Mulli et al. 2001; Urch et al. 2005), but other studies have not (Ibald-Mulli et al. 2004).

Endothelial dysfunction is an initial step in the development of atherosclerosis (Quyyumi 1998), and an important factor in the development of hypertension and heart failure (Corretti et al. 2002). Impaired endothelium-dependent vascular reactivity has been linked epidemiologically to unstable angina, myocardial infarction, and cardiovascular deaths, even in the absence of obstructive coronary artery disease (Halcox et al. 2002). Forearm venous plethysmography is a well-established method for assessing systemic vascular function, with defined accuracy and reproducibility (Joannides et al. 2006). The increase in flow after infusion of acetylcholine is considered by many to be the “gold standard” for measuring endothelial function, and is predictive of future cardiovascular events (Heitzer et al. 2001; Perticone et al. 2001). In this study, we chose not to perform acetylcholine infusion during RH to avoid the risk and discomfort of arterial catheterization, and to avoid possible effects on subsequent measurements of RH and plasma NO products. Forearm blood flow during RH correlates well with vasodilation during maximal acetylcholine infusion (Higashi et al. 2001). RH is not primarily endothelium-dependent (Tagawa et al. 1994), but initiates upstream flow-mediated dilatation, which is endothelium-dependent.

The increase in peak flow during RH 3.5 hr after air exposure may be a response to the intermittent exercise during exposure. In a recent study of 12 sedentary subjects (Bousquet-Santos et al. 2005), forearm blood flow during RH increased 10 and 60 min after 10 min of maximal exercise, and returned to baseline levels 120 min after exercise. Our subjects required some degree of fitness to perform 2 hr of intermittent exercise; it is possible that the increased fitness level of our subjects, together with the prolonged intermittent exercise, caused a more delayed and persistent increase in peak flow. It is well established that chronic exercise improves endothelial function (Clarkson et al. 1999; Rakobowchuk et al. 2005). Diurnal variation could also be responsible for the increase at 3.5 hr after exposure, because this measurement was performed in the afternoon. In contrast, all other measurements were performed earlier in the day. Artifact introduced by order of exposure or participant bias was prevented by the double-blind, randomized, crossover study design. Although the reason for the delayed increase in peak flow after air exposure is not entirely clear, exposure to UFP appeared to block the effect.

We measured venous concentrations of nitrate and nitrite as products of NO metabolism. Nitrate levels decreased significantly after UFP, relative to air exposure. Reductions in venous nitrate levels immediately after UFP exposure correlated significantly with the reductions in peak forearm blood flow 3.5 hr after exposure (Figure 5), although changes in nitrate levels at other times did not correlate with forearm flow.

NO, produced by endothelial cells from l-arginine via NO synthase, mediates vasodilation in both pulmonary and systemic vascular beds (Ikeda et al. 1995; Joannides et al. 1995). The generation of NO is driven by an influx of calcium via calcium-activated potassium channels, which activate endothelial NO synthase (Cooke et al. 1991), and by calcium-independent mechanisms associated with blood flow shear stress (Fleming et al. 1998). However, RH is governed by other factors in addition to NO; infusion of an NO synthase inhibitor into the forearm circulation only partially blocks RH. Furthermore, the peak flow response during RH does not appear to be mediated by NO, and may represent a response to tissue hypoxia, adenosine accumulation, vasodilating prostaglandins, myogenic factors, or endothelium-dependent hyperpolarizing factor (Tagawa et al. 1994). Because NO does not appear to be important in regulating peak flow during RH, it seems unlikely that the UFP effects on peak flow observed in our study are directly related to UFP effects on NO.

Inhalation of UFP may deplete NO by delivering reactive chemical species to the vascular endothelium. When compared on a particle mass basis, UFP have greater oxidant capacity than larger particles (Brown et al. 2001; Li et al. 2003), possibly related to their high surface area and potential to adsorb reactive chemical species from the air. Furthermore, UFP can cross cell membranes via diffusional mechanisms, entering cell nuclei and mitochondria (Geiser et al. 2005). UFP and their burden of reactive chemical species may enter the pulmonary vascular space and then the systemic circulation, although the degree to which this occurs in humans is unclear (Mills et al. 2006; Nemmar et al. 2002). Reactive oxygen species formed as a result of particle exposure may react with NO, forming peroxynitrite, in turn perpetuating the oxidant injury that contributes to endothelial dysfunction and atherosclerosis.

Other mechanisms may be involved, including lung inflammatory responses with secondary vascular effects. However, our previous studies have found no evidence for airway inflammation, as measured by sputum induction and exhaled NO, in response to carbon UFP exposure (Frampton et al. 2004; Pietropaoli et al. 2004b, 2004c). In a recent study, recovery of inflammatory cells in bronchoalveolar lavage fluid did not increase 18 hr after exposure to concentrated ambient UFP (Samet et al. 2007). Thus airway inflammation is unlikely to explain the vascular effects of UFP exposure.

Epidemiologic, panel, and other clinical studies also support the hypothesis that exposure to particulate matter, including UFP, alters endothelial function. In a large panel study of people with diabetes (O’Neill et al. 2005), several different measures of PM exposure were significantly associated with reductions in vascular reactivity. Recently, Künzli et al. (2005) found a significant relationship between long-term PM exposure and a marker of atherosclerosis, the thickness of the carotid artery endothelium measure by ultrasound. Short-term inhalation of concentrated ambient particles plus ozone caused systemic vasoconstriction in resting human subjects (Brook et al. 2002), and the effect appeared to be most strongly related to the organic and elemental carbon content of the particles (Urch et al. 2004). Inhalation of diesel exhaust for 1 hr, with intermittent exercise, attenuated the forearm vasodilation response to infusion of both acetylcholine and nitro-prusside, but not verapamil, in human subjects, both 2 and 6 hr after exposure (Mills et al. 2005). This suggests that diesel exhaust, which is rich in UFP and elemental carbon, impairs both endothelium-dependent and -independent vasodilation. However, the diesel studies used PM concentrations approximately 6-fold higher (∼300 μg/m^3^) than our study (50 μg/m^3^), and included diesel exhaust gases, particularly nitrogen dioxide and CO.

*In vitro* and *in vivo* animal studies also provide evidence of a relationship between particle inhalation and endothelial dysfunction. For example, Batalha et al. (2002) reported that concentrated ambient particles induced vasoconstriction of small pulmonary arteries in rats. Instillation of residual oil fly ash increased pulmonary artery pressure in rats, via mechanisms involving epidermal growth factor (Huang et al. 2002). Urban particles caused constriction of rat pulmonary artery rings, via the angiotensin-I receptor (Li et al. 2005). Furthermore, extracts of particles from motorcycle exhaust enhanced vasoconstriction *in vitro*, via pathways involving reactive oxygen species and calcium flux (Tzeng et al. 2003).

We have shown previously that exposure to carbon UFP with intermittent exercise causes a significant decrease in the pulmonary diffusion capacity for CO (Pietropaoli et al. 2004b), and reduces peripheral blood leukocyte expression of some adhesion molecules (Frampton et al. 2006). Both of these effects may result from pulmonary vasoconstriction. Effects on peripheral blood leukocytes were generally maximal at 3.5 hr after exposure—similar to the timing of effects on RH in the present study. Taken together, our data suggest that inhalation of carbon UFP with intermittent exercise alters both pulmonary and systemic vascular function.

There are limitations to this study. Effects of breathing laboratory-generated carbon UFP may not be representative of ambient UFP, although ambient UFP contain elemental carbon. The mass concentration of particles used in this study (50 μg/m^3^) and the particle number concentrations (∼10^7^/cm^3^) exceed those encountered in most outdoor environments, although particle numbers on major highways can reach peaks of 10^7^ particles/cm^3^ (Kittelson et al. 2004). The subjects were healthy lifetime nonsmokers, without significant cardiovascular disease, and do not represent those most susceptible to the cardiovascular effects of PM exposure. Exposures were by mouthpiece, which bypasses nasal clearance mechanisms and alters breathing patterns. However, UFP in the 20- to 40-nm size range are predicted to have relatively low nasal deposition (Daigle et al. 2003), so oral–nasal breathing would not be expected to substantially reduce UFP pulmonary deposition in these studies. Finally, our study, like most human controlled exposure studies, included a relatively small number of subjects, which limited the statistical power. However, the observation of effects on both vascular function and NO metabolism provide biologic plausibility to our findings.

The observed effects are small and not clinically important for healthy subjects. However, for patients with critical coronary artery disease, small changes in vascular responsiveness combined with increased myocardial oxygen demands could worsen ischemia and precipitate adverse events. For example, in a recent clinical study (Mills et al. 2007), exposures to diluted diesel exhaust increased the myocardial ischemic burden in men with stable coronary artery disease and previous myocardial infarction. Based on our initial findings in healthy volunteers, it seems highly plausible that inhalation of UFP would present risks similar to that for diesel exhaust in patients with coronary artery disease. Taken together with other human and animal studies of PM exposure, there is increasingly compelling evidence that inhalation of PM impairs systemic endothelial function. Our findings support a role for UFP in the vascular effects of PM exposure.

## Conclusion

In comparison with air exposure, inhalation of 50 μg/m^3^ carbon UFP reduced peak hyperemic forearm blood flow, increased minimal vascular resistance, and reduced plasma nitrate concentrations. These findings support the hypothesis that inhalation of UFP impairs systemic vascular function and reduces NO bioavailability. These effects represent potential mechanisms linking particle exposure and adverse cardiovascular effects.

## Figures and Tables

**Table 1:** Vital signs before (baseline) and after exposure (mean ± SE).

Exposure	Time	Oxygen saturation (%)	HR (beats/min)	Systolic BP (mmHg)	HR × BP
Air	Baseline	97.4 ± 0.2	77.0 ± 3.5	125.4 ± 3.1	9,618 ± 451
	0 hr	97.0 ± 0.2	94.8 ± 4.2	122.5 ± 3.0	11,494 ± 399
	3.5 hr	97.4 ± 0.3	77.8 ± 2.8	116.4 ± 3.7	9,027 ± 391
	21 hr	97.4 ± 0.2	78.6 ± 2.9	119.2 ± 2.7	9,315 ± 305
	45 hr	97.6 ± 0.3	81.8 ± 2.6	120.8 ± 2.9	9,815 ± 257
UFP	Baseline	97.5 ± 0.3	76.4 ± 3.8	127.9 ± 3.3	9,770 ± 537
	0 hr	97.0 ± 0.2	96.8 ± 4.8	125.7 ± 4.2	12,108 ± 613
	3.5 hr	97.4 ± 0.3	81.1 ± 2.9	119.6 ± 3.1	9,666 ± 365
	21 hr	97.3 ± 0.3	79.9 ± 2.7	120.4 ± 4.4	9,569 ± 413
	45 hr	97.6 ± 0.3	79.1 ± 2.7	121.6 ± 3.6	9,565 ± 342
